# Higher rate of undetected intraoperative damage of latex-free surgical gloves worn by scrub nurses

**DOI:** 10.1186/s13741-025-00539-3

**Published:** 2025-05-06

**Authors:** Leon Euler-Schmidt, Artur Barsumyan, Jan Adriaan Graw, Christian Soost, Yvonne Stephan, Rene Burchard

**Affiliations:** 1 Department of Health, THM Technical University Mittelhessen, Giessen, Germany; 2 Philipps-University of Marburg, Marburg, Germany; 3 Department of Anesthesiology and Intensive Care Medicine, Ulm University Hospital, Ulm, Germany; 4 University of Siegen, Siegen, Germany; 5 Department of Orthopaedics and Traumatology, University Hospital of Giessen and Marburg, Marburg, Germany; 6 Department of Orthopaedics and Trauma Surgery, Lahn-Dill-Kliniken, Dillenburg, 35683 Germany

**Keywords:** Surgical gloves, Intraoperative perforation, Infection prevention, Latex

## Abstract

**Background:**

Surgical gloves are a medical product and a cornerstone of prevention from surgical site infections and staff injury. This study aimed to investigate the integrity of surgical gloves worn by scrub nurses during selected procedures in both general and trauma surgery. The frequency of defects such as perforations or tears was identified. Furthermore, differences in durability between latex and latex-free gloves were analyzed.

**Methods:**

In a 3-month period, a total of 139 surgical glove pairs, both latex and latex-free, used during general or trauma surgery in an academic teaching hospital were collected immediately after procedures. The gloves were subjected to watertightness testing following European norm ISO EN 455:2022 standards. Only gloves visually presumed to be intact were tested for any concealed perforations.

**Results:**

The number of perforated glove pairs was similar in both departments (general surgery 25% (*n* = 14 of 57) vs. trauma surgery 28% (*n* = 23 of 82), *p* = 0.79). However, differences in perforation rates by glove models (latex vs. latex-free) were noticed. The likelihood of perforation was increased by a factor of 4.24 with the use of latex-free gloves (χ^2 = 8.48, *p* = 0.004).

**Conclusions:**

Perforation of surgical gloves worn by scrub nurses is a common event during various surgical procedures in general and trauma surgery. In several cases, members of the surgical team do not notice a perforation of a glove. The risk of undetected damage to a surgical glove is significantly higher when latex-free gloves are used. Further research is needed to investigate if the use of a second layer of gloves could reduce this perioperative risk for surgical staff and patients.

## Background

Surgical site infections (SSIs) are healthcare-acquired infections and remain among the most serious surgical complications (Ansari et al. 2019). They are associated with an 11 times higher risk of death and an average prolonged hospital stay of 10 days (Matsuda et al. 2023).

Surgical gloves are a crucial element of personal protective equipment worn by healthcare workers such as scrub nurses and surgeons during surgery and medical interventions (Levy et al. 2016). They act as a barrier, protecting both the patient from potential pathogens carried by the surgeons’ or scrub nurses’ hands and the staff from exposure to the patient’s bodily fluids. Maintaining sterility throughout a surgical procedure is paramount to prevent SSIs with the associated significant increase in patient morbidity and mortality (Misteli et al. 2009; Kolasiński 2018). Representing the largest bidirectional migration barrier for microorganisms, the concept of using surgical gloves during interventional procedures required that this barrier function remains intact (Simone et al. 2020; Harnoss et al. 2010). Surgical gloves are certified medical products and must therefore meet the highest safety standards. In the European Union, these comprehensive safety requirements must be met in accordance with the standards defined by the European Committee for Standardization EU 2017/745 (CEN 2024; Union and Verordnung (EU) 2017).

Traditionally, surgical gloves were manufactured from latex (Rego and Roley 1999). However, concerns regarding latex allergies among healthcare workers have led to a widespread shift towards the use of latex-free alternatives such as nitrile and vinyl gloves (Hunt et al. 2002; Enz et al. 2021). While latex-free gloves offer a solution for those with latex allergies, there is growing evidence that they may have some drawbacks compared to their latex counterparts (Aldlyami et al. 2010).

While previous research on glove perforation has focused primarily on surgeons during specific procedures, there is a limited body of research that specifically addresses the surgical glove integrity of gloves used by scrub nurses (Oliveira and Gama 2016). A systematic literature review revealed no specific data on perforation rates of surgical gloves used by scrub nurses during common surgical procedures, although the scrub nurse is an important member of the surgical team (Lee et al. 2015 Mar; Guanche Garcell et al. 2022; Thomson et al. 2022; Al-Maiyah et al. 2005; Matsuoka et al. 2022; Timler et al. 2014). It requires a high level of training and knowledge around the highly specialized settings in operating theaters (San Martin-Rodriguez et al. 2019). The nurse has a thorough understanding of the surgical procedures and directly assists one or more surgeons when they perform the surgery. Additionally, the nurse must prepare the next surgical steps and identify intraoperative needs (Mitchell and Flin 2008).

Therefore, the aim of this study was to investigate unnoticed perforations in surgical gloves worn by scrub nurses during selected surgical procedures in trauma and general surgery. Occurrence of glove damage according to the ISO EN 455–1 was defined as the primary endpoint (CEN 2024). In addition, the presented study aimed to identify potential differences in the frequency of perforations of differently manufactured surgical gloves (latex vs. latex-free).

## Methods

### Study design and experimental setup

This single-center prospective clinical study was performed in the operation theater of a German academic teaching hospital. All general and trauma surgeries with open, laparoscopic, arthroscopic, osteosynthetic, endoprosthetic, and vascular procedures during a period of 3 months (06/2023 – 08/2023) were included. In case of double gloving, only the outer gloves were collected, and changing gloves during the surgical procedure led to exclusion from the study. Only gloves that were recognized as intact by the surgical staff were collected. Gloves were only collected during aseptic procedures to reduce the risk to laboratory scientists of spreading pathogens through contaminated or blood-stained gloves. The gloves were collected after each individual operation and placed in an individual plastic bag that was labeled with all relevant data. Because trainees might use tools inappropriately with consecutive perforation risk and to prevent therefore bias of staff qualifications, only used gloves used by experienced and board-certified scrub nurses were included. In accordance with the requirements of the ethics committee, data were anonymized immediately after the gloves were collected.

During the surgical procedures, commercially available sterile, powder-free, disposable surgical gloves sizes 6 to 8 were used, either made of latex (Signature® Latex Essential, Medline Industries Inc., Northfield, IL, USA) or latex-free (Sensi-Care® PI, Medline Industries Inc., Northfield, IL, USA). In addition, both glove models have the same manufacturer specifications, in particular the identical material thickness of 0.23 mm. All glove samples were inspected for undetected perforations within 24 h. Care was taken to ensure that the time period remained comparable for all gloves; the laboratory scientists consistently tested the gloves on the following day in the same order as they were collected on the day of surgery. During this period, the nurse prevented the surgical gloves from sticking together by removing blood residues from the glove with saline solution because sticking and pulling apart the gloves during evaluation could potentially have caused additional damages.

**Fig. 1:**
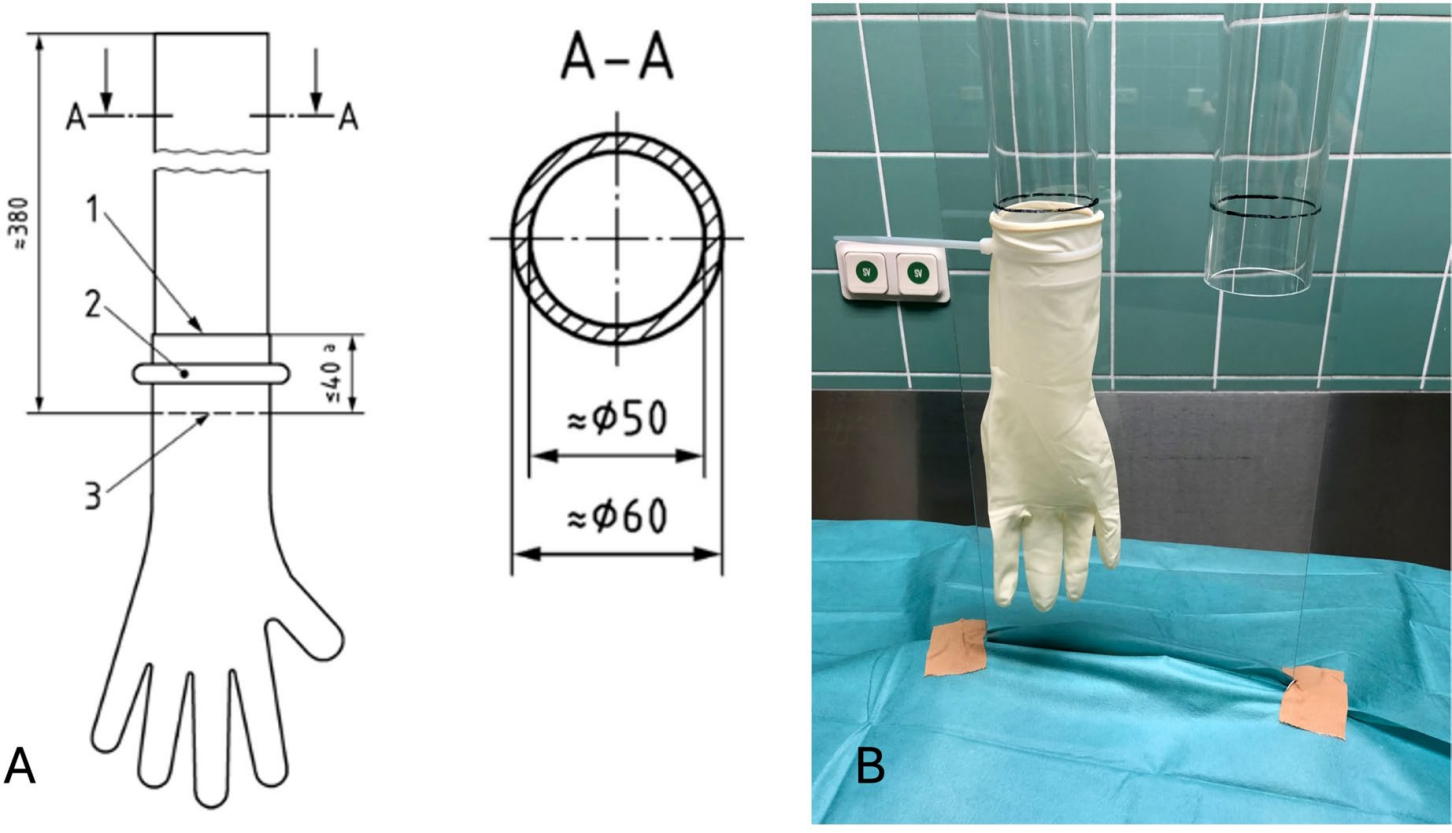
Watertightness tube system to find perforations and tears via freedom from holes testing for used medical surgical gloves. **A** Technical drawing (data in millimeters) of the waterproofing pipe in accordance with ISO EN 455–1:2022. (En 2000). **B** Self-made testing system watertightness tube made from acrylic plastic with two cylinders with an outside diameter of 60 mm and an inside diameter of 50 mm

The examination of tears and micro perforations of the surgical gloves was executed within 24 h by applying the freedom from holes testing method described in the ISO EN 455–1:2022 (medical gloves for single use, Part 1: Requirements and testing for freedom from holes, watertightness test) (Fig. 1a). A specifically manufactured watertightness measuring setup was made from two acrylic cylinders that have an outer diameter of 60 mm. The gloves were stretched over each of the cylinders up to a maximum of 40 mm and attached with a nylon cable to avoid slipping (Fig. 1b).

After successful attachment, the surgical gloves were carefully filled with 1000 ml (± 50 ml) of warm water (15–25 °C) in accordance with the specifications of ISO EN 455–1:2022. A commercially available measuring cup with a capacity of 1000 ml was used. After 3 min, the surgical glove was deflated, and the process described was repeated.

### Ethical approval and informed consent

The ethics committee of Philipps University of Marburg approved the present study according to the ethical standards (number of ethical approval: 24–130 ANZ). Written informed consent of included specimen was waived by the ethics committee because of the anonymous nature of the presented study. All methods were performed in accordance with relevant guidelines and regulations as stated in the Helsinki Declaration.

### Statistical analysis

A power analysis was conducted with the software package G*Power® to compute the a priori required sample size for the chi-square test of independence with a minimum sample size of *n* = 88.

Data were analyzed using the open-source software R (R Core Team (2013), R: a language and environment for statistical computing, R Foundation for Statistical Computing, Vienna, Austria). The frequency analysis was carried out using the chi-square test of independence and the specification of odds ratios. A multivariable analysis was performed using a logistic regression model due to the categorical dependent variable. The following covariates were included in the multivariable analysis: type of surgical glove, duration of the procedure, and type of procedure. The association between these variables and glove perforation rate was then examined. The selection of these covariates was based on clinical relevance and potential influence on glove perforation. The inclusion of these variables in the study was intended to adjust for confounding factors and provide a more accurate estimate of the relationship between glove type and perforation rates.

## Results

During a 3-month period, 155 surgical glove pairs were included in the study. To reduce bias induced by much longer than average operations, because long surgical procedures are more likely to lead to perforation of the surgical gloves, all cases greater than 1.5 times the IQR (> 140 min) of the variable surgery duration were considered outliers. Thereby, the sample size was reduced to *n* = 139 glove pairs. The total wearing time of the surgical gloves during procedures in the general surgery department was 8026 min, which corresponds to approximately 134 h. In the trauma surgery department, surgical gloves were worn for a total of 4470 min (approximately 74.5 h) and 3556 min in general surgery (approximately 59 h). This corresponds to an average wearing time of 54.51 min per pair of gloves in trauma surgery and 62.39 min in general surgery (*p* = 0.11). Further descriptive statistics of the variables can be found in Table 1..

**Table 1.** Occurrence of perforation, type of surgical procedure, and used type of gloves

	*n*	%
Perforation		
* Yes*	37	27%
* No*	102	73%
Type of procedure		
* Arthroscopic*	12	9%
* Endoprosthetic*	25	18%
* Vascular surgery*	9	7%
* Laparoscopic*	31	22%
* Laparotomic*	17	12%
* Osteosynthetic*	45	32%
Surgery glove type		
* Latex*	52	37%
* Latex-free*	87	63%
Surgery duration *(minutes)*	Mean 57.74	SD 27.32

**Fig. 2:**
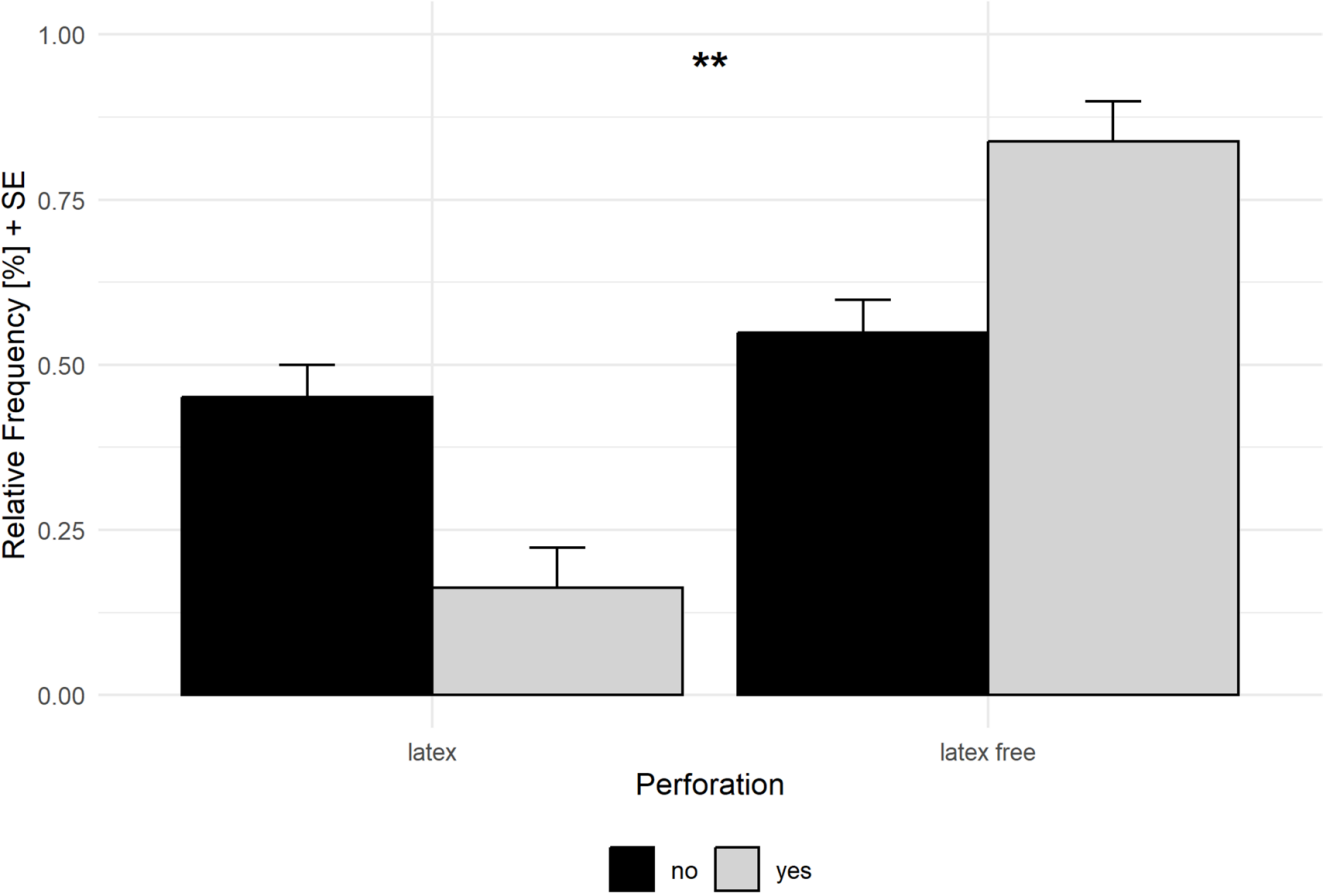
Comparison of perforation frequency between latex and non-latex surgical gloves

The number of perforations was quite similar in both departments (general surgery 25% (*n* = 14 of 57) vs. trauma surgery 28% (*n* = 23 of 82), *p* = 0.79). However, the differences in perforated glove models (latex vs. latex-free) in this study were notable. The chance of perforation was increased by a factor of 4.24 with the use of latex-free gloves (χ^2 = 8.48, *p* = 0.004) (Fig. 2).

An additional multivariable analysis, controlling for the duration of surgery and the type of procedure, confirmed these findings (Table 2). Table 2 shows the results of the logistic regression. Column 3 of the table shows the effect coefficients as odds ratios. The odds of perforation is 4.13 times higher with latex-free gloves compared to latex gloves (*p* < 0.01). In addition, an interesting result of the control variable can be observed. The odds of perforation increases by 1.036 for every minute longer the procedure lasts (*p* < 0.001). The quality of the regression can be assessed using the pseudo-R2 values. Values of 0.2 to 0.4 indicate an acceptable to very good model fit.

**Table 2:** Logistic regression of grove perforation rates

Dependent variable	Coefficient (SE)	OR (LC, UC)
Constant	− 3.246* (0.936)	
Surgical glove latex-free	1.418** (0.529)	4.128 (1.542, 12.611)
Surgery duration	0.035*** (0.011)	1.036 (1.014, 1.061)
Type of procedure (ref. = arthroscopic)		
Endoprosthetic	− 2.015* (1.137)	0.133 (0.014, 1.409)
Vascular surgery	− 1.674 (1.361)	0.188 (0.012, 2.820)
Laparoscopic	− 1.205 (1.133)	0.300 (0.033, 3.204)
Laparatomic	− 1.193 (1.325)	0.303 (0.013, 3.829)
Osteosynthetic	− 0.431 (0.981)	0.650 (0.102, 5.558)
Log-likelihood	− 64.932	
Cox & Snell *R*^2^	0.201	
Nagelkerke *R*^2^	0.293	
Observations	139	

Standard error in parentheses, *p ≤ .05, **p ≤ .01, ***p ≤ .001. *OR* odds ratio, *SE* standard error

*LC* lower confidence interval, *UC* upper confidence interval

## Discussion

This study demonstrated that glove perforations are a real concern for scrub nurses. All glove perforations detected in this study (27%) remained undetected during procedures, which certainly could increase the risk of wound contamination and transmission of infections. Therefore, factors such as wearing time, surgical procedure, and glove models (latex vs. latex-free) that give a potential impact on the occurrence of hidden glove damages were examined.

De Simone and colleagues showed that a longer wearing time of surgical gloves increases the probability of perforations (Simone et al. 2020). They found a significant loss of 24% of the mechanical resistance of latex gloves after only 30 min of wearing time. The fact that the duration of wearing surgical gloves is significantly related to the risk of perforation was also reported by de Barros and colleagues (de Barros et al. 2021). Accordingly, surgical gloves that were worn for more than an hour had significantly more cracks than gloves that were worn for less than an hour (*p* < 0.001). Sayin and colleagues noted the presence of a direct relationship between the frequency of glove perforations and the duration of the operation, which also corresponds to the data of our study (Sayin et al. 2019).

Furthermore, a different glove perforation rate could be found in assorted surgery departments and in their subdisciplines. In trauma surgery, arthroscopic and endoprosthetic procedures had the lowest perforation rates. In both subdisciplines, over 83% of the surgical gloves remained intact. The lower perforation rate for endoprosthetic procedures in this study in comparison to osteosynthetic procedures appears to be due to standardized glove changes around cementing and before the final implantation, as well as the fact that the procedures are relatively short, with an average surgical glove wear time of 63 min. For arthroscopic procedures, the short duration of use (an average of 20.6 min) might also likely contribute to a lower risk of perforation. The generally high perforation rate for open procedures in trauma surgery can be attributed to the intraoperative handling of sharp-edged subjects such as screws, wires, and sharp bone surfaces, especially in the case of bone fractures. The increased physical force exerted on surgical gloves during trauma surgery procedures, which is caused by handling drills, chisels, saws, and heavy sharp-edged instruments, could lead to more frequent perforations. However, although the absolute and relative number of perforations in osteosynthetic surgery was highest, the rates of surgical glove perforations did not differ statistically compared to the rates in arthroscopic surgery. In this context, the pure operating time might play a relevant role as a cofactor in trauma surgery. The increased risk of perforation in trauma surgery is also described by Goldman and colleagues (Goldman et al. 2016).

In general surgery, the physical stress on surgical gloves plays a rather subordinate role. The 30% perforation rate for laparoscopic procedures can be attributed to the fact that the same surgical glove is worn throughout the procedure, as there is no standardized change for most purely laparoscopic procedures (Matsuoka et al. 2022). In addition, the risk of perforation is increased because the scrub nurse must remove blood and tissue from sharp-pointed endoscopic instruments, which increases the risk of perforation during these tasks. In the study by Laine and Aarnio, a perforation rate of 20% for laparoscopic procedures is already considered to be remarkably high (Laine and Aarnio 2001). In the meta-analysis by Anand and colleagues, the topic is limited exclusively to surgeons, so that no conclusions can be drawn about scrub nurses since their tasks are not comparable (Anand et al. 2022). Other studies suggest that it is precisely the task of cleaning blood and tissue residues by the nurse that can explain a higher perforation rate (Matsuoka et al. 2022; Anand et al. 2022). The authors explain the discrepancy that the surgeon has a lower perforation rate during laparoscopic procedures by the fact that the surgeon’s gloves have little contact with the sharp edges of the instruments, as these are far away from the instrument handle.

Another important finding of this work was a significant difference in perforation rates between the two used glove models (latex vs. latex-free, *p* = 0.004). Similar results were described by Aldlyami and colleagues, who found that latex-free surgical gloves perforated significantly more often than regular latex gloves worn by surgeons (Aldlyami et al. 2010). These results could be confirmed by others with a higher rate of damaged latex-free gloves during endoprosthetic procedures worn by surgeons (Thomas et al. 2011). Although it is unclear if the short contact time during surgical procedures could cause allergic reactions at the patient’s side, most surgical protocols for patients with latex allergy require the removal of all latex gloves, latex products, and latex powder from the operating room (Australian Society of Clinical Immunology and Allergy (ASCIA). Operating suite guidelines for latex allergic patients 2010) On staff’s side, scrub nurses show a higher overall in-gloves time compared to surgeons with a higher impact on potential allergic reactions and glove damages. Nevertheless, there is still a lack of knowledge regarding gloves worn by scrub nurses in particular in an interdisciplinary setting.

In summary, a discussion about the need for higher resistance standards for surgical gloves, as well as new guidelines for the use of double gloves, should be considered, although the WHO sees no evidence for this measure in their 2018 global guidelines for the prevention of surgical site infection guidelines (World Health Organization (WHO). Global guidelines for the prevention of surgical site infection, second edition 2018). However, the effectiveness of double gloving is shown in a meta-analysis by Zhang and colleagues, even though this working group focused on occupational safety aspects (Zhang et al. 2021). Likewise, the National Institute for Health and Care Excellence (UK) states in their work Surgical Site Infections: Prevention and Treatment: “Consider wearing 2 pairs of sterile gloves when there is a high risk of glove perforation, and the consequences of contamination may be serious” (Institute and for Health and Care Excellence (NICE). Surgical site infections: prevention and treatment (NG125) 2019). Beside this, adaptation of the acceptable quality limit (AQL) of 0.65 in the test standard ISO EN 455–1:2022 for the tightness parameter of surgical gloves to the AQL of further medical products that serve to prevent infections should be discussed. For example, contraceptive devices such as condoms must have an AQL of 0.25 and therefore meet significantly higher requirements during production than surgical gloves (e.V. DDIfN. 2015). By bringing greater awareness to this issue, we aim to encourage further research and development of improved glove models (latex vs. latex-free) and detection methods to ensure optimal patient safety during surgical procedures.

### Limitations

This study has several limitations. First, it should be noted that the incidence of SSI and the relationship with perforation were not analyzed because no patient data were collected. Second, the study is limited by its monocentric nature preventing a more universally valid conclusion. A particular limitation in this context is the fact that only one manufacturer of gloves is in use at our center. Further interesting validation of the main findings of this study with gloves from other manufacturers would, therefore, be desirable in the future. A multicenter study would have had the advantage of larger datasets, and thus, more meaningful results could have been published using inferential statistics.

In addition, perforation types were not examined under the microscope, nor was the number of obviously damaged gloves, and therefore excluded from the study, recorded. Furthermore, the test used here may tend to give false-negative results, especially when detecting very small perforations (Walczak et al. 2013). Thus, the perforation rate that was found could even be underestimated.

## Conclusion

Perforation of surgical gloves worn by scrub nurses is a common event during different surgical procedures in general and trauma surgery. In several cases, members of the surgical team do not notice the perforation of gloves. The risk of undetected damage to a surgical glove is significantly higher if latex-free gloves are used. Further research is needed to investigate if the additional and general use of a second layer of gloves could also reduce this perioperative risk for surgical staff and patients.

## Data Availability

No datasets were generated or analysed during the current study.

## Abbreviations

OR: Odds ratio
ISO: International Organization for Standardization
EN: European committee for standardization
SSIs: Surgical site infections
ml: Milliliter
LC: Lower confidence interval
UC: Upper confidence interval
AQL: Acceptable quality level
